# Supplementary material for: Fisetin inhibits Salmonella Typhimurium type III secretion system regulator HilD and reduces pathology in vivo
Source: Microbiol Spectr. 2023 Dec 11;12(1):e02406-23. doi: 10.1128/spectrum.02406-23 (PMC10783070; doi:10.1128/spectrum.02406-23)
Supplement: Supplemental figures — Fig. S1 and S2. [file spectrum.02406-23-s0001.doc]

**
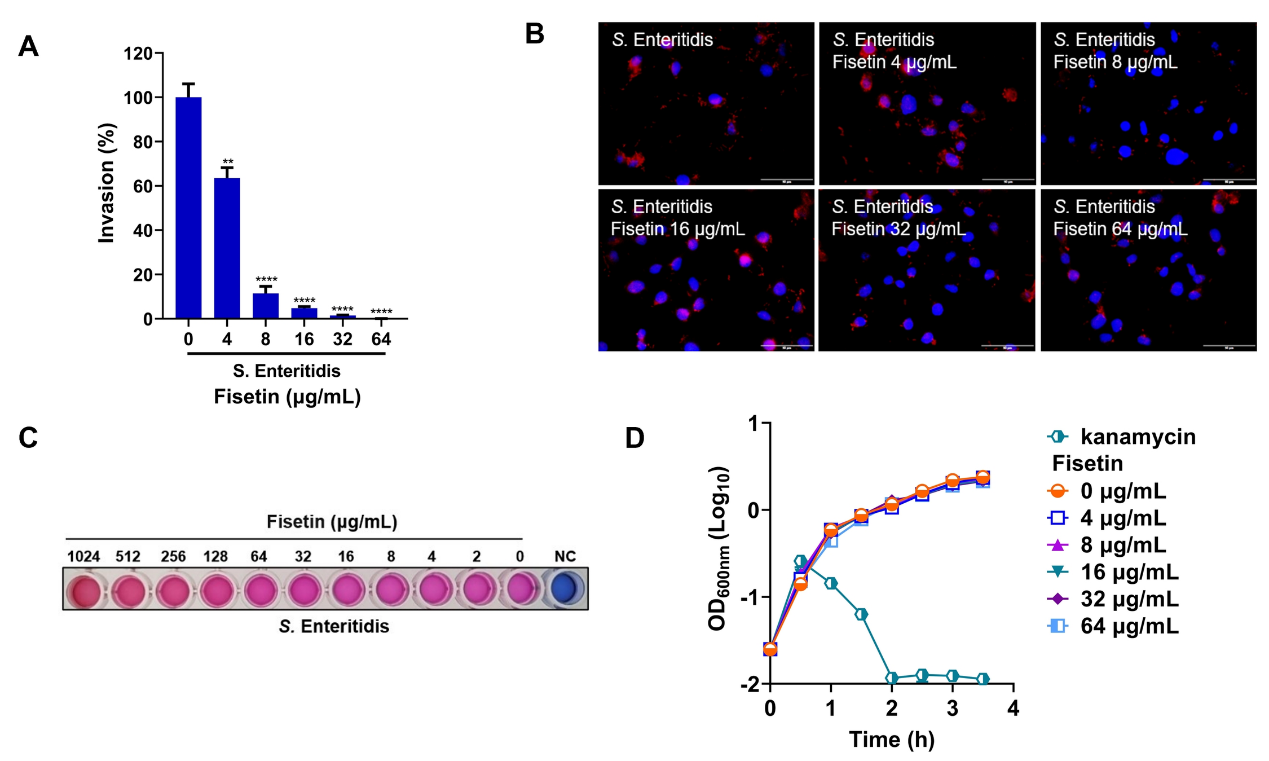
**

**Supplementary Figure 1. Fisetin inhibits *S.* Enteritidis invasion of HeLa Cells.** (**A**) *S.* Enteritidis grown in the presence of fisetin was used to infect HeLa cells, and the percent invasion of the bacteria was determined by the gentamycin protection assay. (**B**) Immunofluorescence images showing the decreased entry of *S.* Enteritidis upon fisetin treatment. (**C**) The MIC of fisetin against *S.* Enteritidis. (**D**) The *in vitro* growth curve of *S.* Enteritidis cultured with different concentrations of fisetin. Data presented in Panel A are the mean ± SD of three independent experiments. Panels A and D are representative of three independent experiments. ****, *P*< 0.0001; **, *P*< 0.01.

**
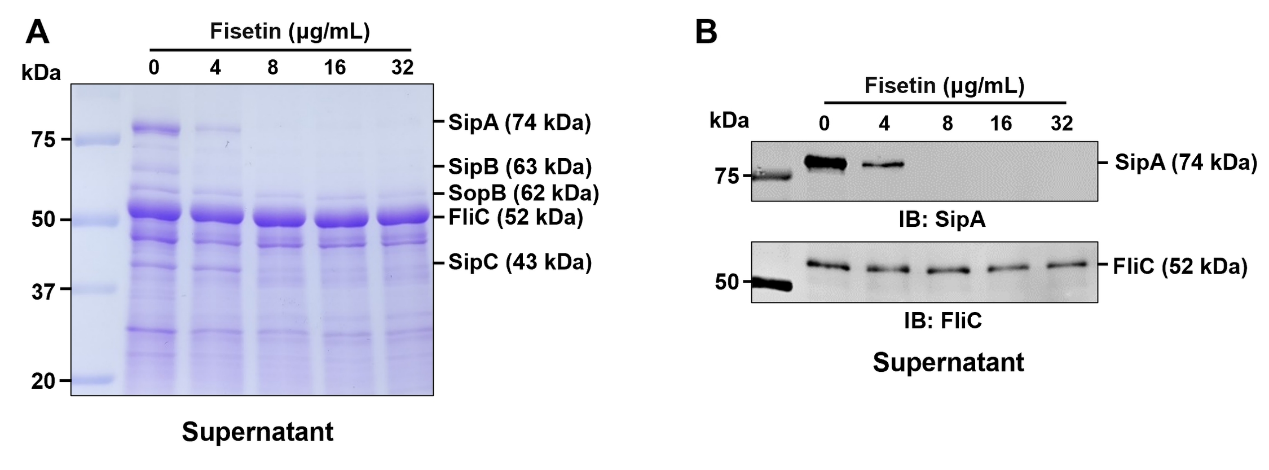
Supplementary Figure 2. Fisetin decreases the secretion of *S.* Enteritidis T3SS-1.** After culturing *S.* Enteritidis with fisetin, the growth media were collected and precipitated by TCA. The effector protein levels in the precipitates were determined by CBB staining (**A**) or western blot using SipA and FliC antibodies (**B**). Data are representative of three independent assays.
