# Supplementary material for: Fisetin inhibits Salmonella Typhimurium type III secretion system regulator HilD and reduces pathology in vivo
Source: Microbiol Spectr. 2023 Dec 11;12(1):e02406-23. doi: 10.1128/spectrum.02406-23 (PMC10783070; doi:10.1128/spectrum.02406-23)
Supplement: Supplemental tables — Tables S1 to S4. [file spectrum.02406-23-s0002.doc]

**Supplementary Table 1. Bacterial strains used in this study**

| Strains | Relevant properties | Source |
| --- | --- | --- |
| *E.coli* strains |  |  |
| DH5α | F^–^ *endA1 glnV44 thi-1 recA1 relA1 gyrA96 deoR nupG purB20* φ80d*lacZ*ΔM15 Δ(*lacZYA-argF*)U169, hsdR17(*rK^–^mK^+^*), λ^–^ | Our collection |
| BL21(Rosetta) | *hsdSB*(*rB^–^mB^–^*) λ(DE3 [*lacI lacUV5-T7p07 ind1 sam7 nin5*]) [*malB^+^*]_K-12_(λ^S^) pLysSRARE[*T7p20 ileX argU thrU tyrU glyT thrT argW metT leuW proL ori*_p15A_](Cm^R^) | Our collection |
| *S.* Typhimurium strains |  |  |
| SL1344 WT | Streptomycin resistant | Cheng et al. ^1^ |
| SL1344Δ*invA* | Deficient in functional T3SS-1 by the deletion of InvA | Cheng et al. ^1^ |
| SL1344 SipA-beta-lactamase (SipA-TEM) | SL1344 carrying the vector of SipA-TEM | This study |
| SL1344Δ*invA* SipA-TEM | SL1344 Δ*invA* carrying the vector of SipA-TEM | This study |
| SL1344 SipB-3×Flag (SipB-3×Flag) | SL1344 chromosomally expressing Flag-tagged SipB | This study |
| SL1344 pJL03-vector | SL1344 carrying the vector of pJL03 | This study |
| SL1344 pJL03-*hilA* | SL1344 carrying the vector of pJL03-*hilA* | This study |
| *S.* Enteritidis | Clinical isolation | This study |

**Supplementary Table 2. PCR primers of genes**

| Gene name | Primer sequence (5’–3’) | Product size (bp) | Source |
| --- | --- | --- | --- |
| *hilA* | F-CTGAGATCTATGCCACATTTTAATCCT  R-CTGGTCGACTTACCGTAATTTAATCAAG | 1680 | This study |
| *hilD* | F-CTGGGATCCATGGAAAATGTAACCTT  R-CTGGTCGACTTAATGGTTCGCCATTT | 948 | This study |
| *prgH* | F-CTGGGATCCATGATTCGTCGATATCTA  R-CTGGTCGACCTATTCATTTGACGATTT | 777 | This study |
| *sipA* | F-CTGGGATCCATGGTTACAAGTGTAAG  R-CTGGTCGACTTAACGCTGCATGTGCA | 2076 | This study |

**Supplementary Table 3. qRT-PCR primers for T3SS-1 structural genes**

| Gene name | Primer sequence (5’–3’) | Product size (bp) | Source |
| --- | --- | --- | --- |
| *prgH* | F-GTTGTGGGCTCGTCAGGTTT  R-CGCTTATTTTCTTCGTTTTCGT | 81 | This study |
| *prgI* | F-AATCTACAAACGCAGGTAA  R-CTGAATAATGGCAGCATC | 168 | This study |
| *prgK* | F-AAAAGGACTGGACCAGGAA  R-AAATCAGGCTCAGCAACG | 123 | This study |
| *invG* | F-GTTTGTTGCGAAAGACGA  R-CCCAGTTGTAGGGAAAGC | 165 | This study |

**Supplementary Table 4.** **The whole sequences of promoters and its PCR primers**

| Gene name | The whole sequences of the promoters (5’–3’) |
| --- | --- |
| *hilA* | 5’-TGCAATGAGGCCAAGTTAAATATGTAAATATTTAGATGCCCGGCGCTGACTCTCTCTGCACCAGGATATACGGCAGCGTCCATTCGATAATCACAGTTAGTTATAACAATATTATTACCAACATGTCAGTTATTTAAAGCACAGGCATAAGCTAAATAATCAAATGTTAAAAACATATAAACCCGAGCCCGTAGAATATGACATTAAGCTCATAATAAAAGCTCAACCTGACCGTTAGTACTAACAGCAGAATTACTGAAACAGTAGATTCTATCCTAACGACTTGTATTAGTTATTATAACTTTTCACCCTGTAAGAGAATACACTATTATCATGCCACATT-3' |
| *hilD* | 5’-ACTATGTATGGCCCTGGGCTTGTTATCGTCTTCTCTTTTGATGTTTCCATATATACTGTTAGCGATGTCTGTCGTTCTCGATAGCAGCAGATTACCGCACAGGACACAGGGATTCCTGATGAAAATAGAATGAAAAGTGAGAAATAAAATCAATTTATTCTGTATAATGCGTCTCAACACATATTAAAAGAACCATCATCCCCATTGGGGCTTAAACTACTGTAGATAAATTACCCAAATTTGGGTTCTTTTGGTGTAACAATCAGACCATTGCCAACACACGCTAATAAAGAGCATTTACAACTCAGATTTTTTCAGTAGGATACCAGTAAGGAACATTAAAATAACATCAACAAAGGGATAATATGGAAAATGTAAC-3’ |

Note: The PCR primers of the promoters are marked in red.

1. Cheng S*, et al.* Identification of a Novel Salmonella Type III Effector by Quantitative Secretome Profiling. *Mol Cell Proteomics* **16**, 2219-2228 (2017).
